# Epidemiologic and Clinical Characteristics of Tubo-Ovarian Abscess, Hydrosalpinx, Pyosalpinx, and Oophoritis in Emergency Department Patients

**DOI:** 10.7759/cureus.11647

**Published:** 2020-11-23

**Authors:** Michael Mohseni, Leslie V Simon, Johnathan M Sheele

**Affiliations:** 1 Emergency Medicine, Mayo Clinic, Jacksonville, USA

**Keywords:** oophoritis, emergency department, sexually transmitted infection, tubo-ovarian abscess, hydrosalpinx, pyosalpinx

## Abstract

Introduction

Pelvic inflammatory disease (PID) is a spectrum of illness ranging from mild illness to more severe forms including tubo-ovarian abscess, hydrosalpinx, pyosalpinx, oophoritis (THPO). The objective of the study was to report rates and clinical characteristics of females presenting to the ED with a diagnosis of THPO in relationship to the presence or absence of sexually transmitted infections (STIs).

Methods

A database of ED patient encounters occurring from April 18, 2014, to March 7, 2017 was created. Analysis of women diagnosed with THPO and who had testing for gonorrhea, chlamydia, or trichomonas by nucleic acid amplification testing or who had a vaginal wet preparation was performed. Patient demographics, ED diagnoses, laboratory tests, medications administered in the ED, and medications prescribed were examined. Categorical variables were summarized as count and percentages and analyzed using the Chi-square test. Continuous variables were summarized as the mean and standard deviation and analyzed using the t-test. All statistical tests were two-sided with a significance level of 0.05.

Results

THPO was diagnosed in 0.3% (56/17,905) of patient encounters. There were 50% (28/56) of women with THPO admitted to the hospital. There were 25.0% (12/48) women who received a positive test result for *Neisseria gonorrhoeae*, *Chlamydia trachomatis*, and/or *Trichomonas vaginalis*. Women with THPO were significantly older, more likely to be infected with gonorrhea, and more likely to be diagnosed with sepsis and PID (P<.05 for all).

Conclusions

THPO is an infrequently encountered entity in the ED. A diagnosis of STI, PID, and sepsis can accompany these presentations. Although an uncommon diagnosis, ED providers must be attentive to patients presenting with pelvic symptoms that could be consistent with THPO to mitigate any complications that may arise and to direct the appropriate treatment.

## Introduction

Pelvic inflammatory disease (PID) is a spectrum of illness that ranges from subclinical or mild illness to more severe forms including tubo-ovarian abscess, hydrosalpinx, pyosalpinx, oophoritis (THPO). Although PID and THPO are more common among women of reproductive age, these entities can also exist in adolescents and in post-menopausal women [1-3]. Patients with these conditions often seek evaluation in the Emergency Department (ED). Clinicians must be attentive to when female patients present with PID symptoms in order to effectively rule in or rule out complications of this disorder. Tubo-ovarian abscesses (TOA), for example, may require surgical intervention to mitigate excessive morbidity and even occasional mortality [4-5].

Although there are frequent case reports of TOA [6-8], only a few large reviews exist in the literature outlining the characteristics of patients with TOA or THPO [9-11]. Furthermore, some of these retrospective reviews occurred several decades ago when epidemiologic and microbiologic patterns of infection were undoubtedly different from more current clinical observations [10-11].

Some of the typical pathogens implicated in PID include *Chlamydia trachomatis*, *Neisseria gonorrhoeae*, and *Trichomonas vaginalis* [5], which are sexually transmitted infections (STIs); PID may also be caused, however, by organisms that are not sexually transmitted. By anatomic extension in the pelvis, THPO pathogenesis involves the spread of inciting microorganisms to adjacent pelvic structures, including but not limited to the Fallopian tubes and ovaries [12]. Severe pelvic infections such as TOA, however, are often polymicrobial in etiology.

The objective of the study was to report the rates and clinical characteristics of females presenting to the ED with a diagnosis of THPO. We aim to describe the overall demographics of the patients as well as relevant clinical findings in relation to the presence or absence of STIs.

## Materials and methods

The study received institutional review board approval from University Hospitals (UH) and Mayo Clinic. A database of 75,000 UH ED patient encounters occurring from April 18, 2014, to March 7, 2017 was created. All patients were ≥18 years of age and received testing for gonorrhea, chlamydia, or trichomonas, or they received a urinalysis and urine culture. Our analysis only included women that were diagnosed with THPO and who had testing for gonorrhea or chlamydia by nucleic acid amplification testing (NAAT), had a NAAT for trichomonas, or who had a vaginal wet preparation performed. Patient demographics, ED diagnoses, laboratory tests, and the medications that were administered in the ED and that were prescribed during the clinical encounter were examined.

Patients were considered to have THPO, sepsis, or PID if they received an ED discharge International Classification of Disease (ICD) 9 or 10 code for that condition at the clinical encounter. For THPO the ICD codes were: 614.0, 614.2, 098.17, 098.37, A54.29, N70, N70.01, N70.02, N70.03, N70.91, N70.92, and N70.93. For sepsis the ICD codes were: 003.1, 038.12, 038.2, 038.8, 098.89, 670.22, 785.52, 995.91, A02.1, A32.7, A39.4, A40, A40.0, A40.1, A40.3, A40.8, A40.9, A41, A41.0, A41.01, A41.1, A41.2, A41.3, A41.4, A41.5, A41.51, A41.52, A41.53, A41.8, A41.81, A41.9, A42.7, A48.3, A54.86, B37.7, O04.87, R65.2, R65.20, R65.21, T81.12, T81.12XA. For PID the ICD codes were: 614.8, 614.9, 616.9, 098.16, 098.36, 099.54, A56.11, A54.24, N73.9, and N74. Patients were considered to be pregnant if they had a positive pregnancy test for received an ICD code of pregnancy: 633.90, 643.93, O00.1, O00.8, O21.9, O00.90, Z32.01, O00, O20, V72.42. Patients were considered infected with *T. vaginalis* if the organism was seen on vaginal wet prep or on urinalysis or there was a positive NAAT. A person was only considered negative for *T. vaginalis* if they had a negative NAAT. Patients were infected with *C. trachomatis* and *N. gonorrhoeae* if they had a positive nucleic acid NAAT (APTIMA, Hologic). Patients were considered infected with an STI if they had a positive test for *C. trachomatis*, *N. gonorrhoeae*, and/or *T. vaginalis*, and uninfected for an STI if they received a negative NAAT for all three organisms.

On urinalysis “trace” protein and ketones were converted to 0.5+ for the analysis while “<2 mg/dL” urobilinogen was converted to 0 mg/dL to treat these as continuous variables for the analysis. Women that received metronidazole in the ED or as an outpatient prescription were considered treated for *T. vaginalis*. Women given ceftriaxone or cefixime plus azithromycin or received a discharge prescription for doxycycline were considered treated for gonorrhea and chlamydia. Missing and erroneous variables were not included in the analysis.

Statistical analysis

Categorical variables were summarized as count and percentages and analyzed using the Chi-square test. Continuous variables were summarized as the mean and standard deviation and analyzed using the t-test. All statistical tests were two-sided with a significance level of 0.05. The analysis was conducted using JMP Pro 14 (SAS Institute Inc., Cary, NC, USA).

## Results

There were 17,905 patient encounters that were analyzed. There were 2.9% (510/17,388) women infected with gonorrhea, 7.8% (1,360/17,381) infected with chlamydia, and 28.7% (1,909/6,660) infected with trichomonas. When examining women that were tested for gonorrhea and chlamydia and who also underwent NAAT to exclude infection with trichomonas there were 44.2% (3,324/7,520) infected with either gonorrhea, chlamydia, and/or trichomonas.

THPO was diagnosed in 0.3% (56/17,905) of patient encounters. Ages for those with THPO ranged from 18-70 years with a mean of 34.6 years. Among the women with THPO there were seven who were married, one who was separated, one who was widowed, one with an unknown marital status, and 46 who were single. Women with THPO were 1.8% (1/56) Asian, 1.8% (1/56) “other”, 8.9% (5/56) White, and 87.5% (49/56) Black/African-American. Among those women with THPO there were none concurrently diagnosed with a Bartholin cyst or abscess, none that were pregnant, six concurrently diagnosed with PID, and two that were concurrently diagnosed with sepsis. Furthermore, 12.5% (7/56) had ≥1 blood culture ordered in the ED and all had no growth.

THPO compared to those without THPO

Table 1 shows the demographic and clinical characteristics between the women with and without THPO. Women with THPO were significantly older, more likely to arrive to the ED by emergency medical services (EMS) or police, have lower emergency severity index score (ESI) (indicating higher triage acuity), more likely to have *T. vaginalis* on vaginal wet prep, more likely to be infected with gonorrhea, have more urine protein, have urine red blood cells (RBCs) ≥10 cells/high powered field (HPF), to be diagnosed with sepsis and PID, and less likely to be pregnant (P<.05 for all).

**Table 1:** Demographic and Clinical Characteristics of Patients with THPO Compared to Those Without THPO Tubo-ovarian abscess, Hydrosalpinx, Pyosalpinx, and Oophoritis (THPO); Emergency Department (ED); Emergency Severity Index (ESI); White Blood Cells (WBC); High Power Field (HPF); Sexually Transmitted Infection (STI)

	+THPO (N= 56)	-THPO (N=17,849)	P-value
Age (years)	34.6 (11.3); 56	28.8 (9.5); 17,849	<0.001
Black/African American race, %	87.5% (49/56)	88.1% (15,663/17,772)	.88
Single-marital status, %	82.1% (46/56)	86.3% (15,397/17,847)	.37
Arrived to ED by EMS/police, %	19.6% (11/56)	7.3% (1,296/17,743)	<0.001
ESI (1-5)	2.9 (.3); 55	3.2 (.5); 17,015	<0.001
Triage pain scale (0-10)	6.2 (3.0); 32	5.2 (3.7); 1,985	.08
ED visit occurred on weekend (vs weekday), %	69.6% (39/56)	70.9% (12,659/17,849)	.83
Hour of ED visit (0-23)	12.9 (5.8); 56	13.6 (5.8); 17,849	.41
Duration of clinical encounter (hours)	2886.8 (3507.8); 56	408.6 (1239.1); 17,841	<0.001
Has a primary care physician (vs no), %	26.8% (15/56)	17.8% (3,168/17,849)	.08
Vaginal wet prep WBCs >10/HPF (vs ≤10)	40.0% (18/45)	32.9% (5,628/17,135)	.31
Vaginal wet prep clue cells, % present	61.5% (24/39)	56.8% (9,476/16,697)	.55
Vaginal wet prep yeast cells, % present	2.8% (1/36)	6.7% (1,105/16,495)	.35
Vaginal wet prep trichomonas, % present	21.6 (8/37)	8.6% (1,414/16,496)	.005
+gonorrhea	10.4% (5/48)	2.9% (505/17,340)	.002
+chlamydia	8.3% (4/48)	7.8% (1,356/17,333)	.90
+trichomonas	45.0% (9/20)	28.6% (1,900/6,640)	.11
+any STI	57.1% (12/21)	44.2% (3,312/7,499)	.23
Urine bacteria (0-4+)	0.8 (1.0); 40	1.0 (1.1); 11,462	.23
Urine protein (0-3+)	0.7 (.9); 52	0.4 (.7); 15,793	.009
Urine RBCs ≥10 cells/HPF (vs <10)	43.9% (18/41)	29.7% (3,404/11,466)	.047
Urine urobilinogen (0-12mg/dL)	1.0 (1.9); 53	.7 (1.4); 15,815	.18
Urine leukocyte esterase (0-3+)	.8 (1.1); 51	.8 (1.1); 15,596	.97
Urine WBCs ≥10 cells/HPF (vs <10)	50.0% (20/40)	41.5% (4,755/11,468)	.27
Urine nitrite, % present	0% (0/53)	3.9% (617/15,811)	.14
Urine blood (0-3+)	.9 (1.2); 51	.8 (1.1); 15,584	.52
Urine bilirubin (0-3+)	.2 (.7); 53	.05 (.3); 15,802	.05
Urine ketones (0-3+)	.3 (.6); 53	.2 (.5); 15,775	.19
Diagnosed with sepsis, % yes	3.6% (2/56)	.1% (23/17,849)	<0.001
Diagnosed with pelvic inflammatory disease, % yes	10.7% (6/56)	2.3% (409/17,849)	<0.001
+Pregnant	0% (0/56)	21.2% (3,791/17,849)	<0.001

Admitted versus discharged patients with THPO

There were 50% (28/56) of women with THPO admitted to the hospital. Patients admitted to the hospital were significantly younger than those who were not admitted (Table 2). There were no significant differences between those admitted and not admitted for examined triage and demographic variables, vaginal wet preparation findings, or differences on the urinalysis. Women admitted with THPO were not more likely to be diagnosed with sepsis or PID than those with THPO and not admitted.

**Table 2:** Women with THPO admitted from the ED compared to those with THPO discharged from the ED Tubo-ovarian abscess, Hydrosalpinx, Pyosalpinx, and Oophoritis (THPO); Emergency Department (ED); Emergency Severity Index (ESI); White Blood Cells (WBC); High Power Field (HPF); Sexually Transmitted Infection (STI)

	+THPO admitted from ED (N= 28)	+THPO not admitted from ED (N=28)	P-value
Age (years)	31.4 (8.8); 28	37.9 (12.6); 28	.03
Black/African American race, %	92.9% (26/28)	82.1% (23/28)	.23
Single-marital status, %	85.7% (24/28)	78.6% (22/28)	.49
Arrived to ED by EMS/police, %	10.7% (3/28)	28.6% (8/28)	.09
ESI (0-5)	2.9 (.3); 28	3.0 (.2); 27	.32
Triage pain scale (0-10)	6.1 (3.1); 28	7.3 (2.4); 4	.42
Has a primary care physician (vs no), %	28.6% (8/28)	25.0% (7/28)	.76
Vaginal wet prep WBCs >10/HPF (vs ≤10)	50.0% (12/24)	28.6% (6/21)	.14
Vaginal wet prep clue cells, % present	35.0% (7/20)	42.1% (8/19)	.65
Vaginal wet prep yeast cells, % present	5.6% (1/18)	0% (0/18)	.31
Vaginal wet prep trichomonas, % present	21.1% (4/19)	22.2% (4/18)	.93
+gonorrhea	7.7% (2/26)	13.6% (3/22)	.50
+chlamydia	11.5% (3/26)	4.5% (1/22)	.38
+trichomonas	35.7% (5/14)	66.7% (4/6)	.20
+any STI	46.7% (7/15)	83.3% (5/6)	.13
Urine protein (0-3+)	.6 (.8); 28	.9 (1.0); 24	.18
Urine WBCs ≥10 cells/HPF (vs <10)	52.2% (12/23)	47.1% (8/17)	.75
Urine RBCs ≥10 cells/HPF (vs <10)	39.1% (9/23)	50.0% (9/18)	.49
Urine bilirubin (0-3+)	.1 (.4); 28	.4 (.9); 25	.15
Urine leukocyte esterase (0-3+)	.9 (1.1); 26	.8 (1.1); 25	.79
Diagnosed with sepsis, % yes	3.6% (1/28)	3.6% (1/28)	1.0
Diagnosed with pelvic inflammatory disease, % yes	17.9% (5/28)	3.6% (1/28)	.08

THPO infected and uninfected with gonorrhea

There were 10.4% (5/48) of women infected with *N. gonorrhea*. Women with THPO and gonorrhea, compared to those with THPO and no gonorrhea, were significantly younger, more likely to have *T. vaginalis* on vaginal wet prep, more likely to have a positive test for *T. vaginalis*, and more likely to be infected with chlamydia (P≤.02 for all) (Table 3).

**Table 3:** Women with THPO and Gonorrhea Compared to Those with THPO and No Gonorrhea Tubo-ovarian abscess, Hydrosalpinx, Pyosalpinx, and Oophoritis (THPO); Emergency Department (ED); Emergency Severity Index (ESI); White Blood Cells (WBC); High Power Field (HPF)

	+THPO and +gonorrhea (N=5)	+THPO and -gonorrhea (N=43)	P-value
Age (years)	25.2 (2.4); 5	33.1 (8.5); 43	<0.001
Black/African American race, %	100% (5/5)	86.1% (37/43)	.37
Arrived to ED by EMS/police, %	40.0% (2/5)	11.6% (5/43)	.09
ESI (0-5)	3 (0); 5	3.0 (.2); 42	.16
Vaginal wet prep WBCs >10/HPF (vs ≤10)	60.0% (3/5)	36.8% (14/38)	.32
Vaginal wet prep clue cells, % present	40.0% (2/5)	38.2% (13/34)	.94
Vaginal wet prep yeast cells, % present	0% (0/4)	3.1% (1/32)	.72
Vaginal wet prep trichomonas, % present	80.0% (4/5)	12.5% (4/32)	<0.001
+Chlamydia	40.0% (2/5)	4.7% (2/43)	.007
+Trichomonas	100% (4/4)	33.3% (5/15)	.02
Urine protein (0-3+)	.8 (.8); 5	.7 (.9); 40	.72
Urine WBCs ≥10 cells/HPF (vs <10)	80.0% (4/5)	41.9% (13/31)	.17
Urine leukocyte esterase (0-3+)	1.8 (1.0); 4	.7 (1.0); 39	.11
Diagnosed with sepsis, % yes	0% (0/5)	2.3% (1/43)	.73
Diagnosed with pelvic inflammatory disease, % yes	0% (0/5)	11.6% (5/43)	.42

THPO infected and uninfected with chlamydia

There were 9.1% (4/44) of women with THPO that were infected with *C. trachomatis*. Women with THPO and chlamydia, compared to those with THPO and no chlamydia, were significantly younger and more likely to also be infected with gonorrhea (P<.01 for all) (Table 4).

**Table 4:** Women with THPO and Chlamydia Compared to Those with THPO and No Chlamydia Tubo-ovarian abscess, Hydrosalpinx, Pyosalpinx, and Oophoritis (THPO); Emergency Department (ED); Emergency Severity Index (ESI); White Blood Cells (WBC); High Power Field (HPF)

	+THPO and +chlamydia (N=4)	+THPO and -chlamydia (N=44 )	P-value
Age (years)	22.5 (3.3); 4	33.1 (8.2); 44	.001
Black/African American race, %	100% (4/4)	86.4% (38/44)	.43
Arrived to ED by EMS/police, %	25.0% (1/4)	13.6% (6/44)	.54
ESI (0-5)	3 (0); 4	3.0 (.21); 43	.16
Vaginal wet prep WBCs >10/HPF (vs ≤10)	75.0% (3/4)	35.9% (14/39)	.13
Vaginal wet prep clue cells, % present	25.0% (1/4)	40.0% (14/35)	.56
Vaginal wet prep yeast cells, % present	0% (0/4)	3.1% (1/32)	.72
Vaginal wet prep trichomonas, % present	25.0% (1/4)	21.2% (7/33)	.86
+gonorrhea	50.0% (2/2)	6.8% (3/44)	.007
+trichomonas	50.0% (1/2)	47.1% (8/17)	.94
Urine protein (0-3+)	1.3 (1.0); 4	.6 (.8); 41	.28
Urine WBCs ≥10 cells/HPF (vs <10)	75.0% (3/4)	43.8% (14/32)	.24
Urine leukocyte esterase (0-3+)	1.8 (1.0); 4	.7 (1.0); 39	.11
Diagnosed with sepsis, % yes	0% (0/4)	2.3% (1/44)	.76
Diagnosed with pelvic inflammatory disease, % yes	25.0% (1/4)	9.1% (4/44)	.37

THPO infected and uninfected with trichomonas

Among women with THPO and who had a positive test for *T. vaginalis* or who had a positive*T. vaginalis* NAAT there were 45.0% (9/20) of women were infected with *T. vaginalis*. Women with THPO and trichomonas, compared to those with THPO and no trichomonas, were more likely to be infected with gonorrhea (P≤.02) but not chlamydia (P=.94) (Table 5).

**Table 5:** Women with THPO and Trichomonas Compared to Those with THPO and No trichomonas Tubo-ovarian abscess, Hydrosalpinx, Pyosalpinx, and Oophoritis (THPO); Emergency Department (ED); Emergency Severity Index (ESI); White Blood Cells (WBC); High Power Field (HPF)

	+THPO and +trichomonas (N=9)	+THPO and -trichomonas (N=11)	P-value
Age (years)	27.4 (3.7); 9	32.5 (9.5); 11	.13
Black/African American race, %	88.9% (8/9)	100% (11/11)	.26
Arrived to ED by EMS/police, %	33.3% (3/9)	27.3% (3/11)	.77
ESI (0-5)	2.9 (.3); 9	3 (0); 10	.35
Vaginal wet prep WBCs >10/HPF (vs ≤10)	55.6% (5/9)	36.4% (4/11)	.39
Vaginal wet prep clue cells, % present	55.6% (5/9)	33.3% (3/9)	.34
Vaginal wet prep yeast cells, % present	0% (0/8)	0% (0/9)	NA
Vaginal wet prep trichomonas, % present	88.9% (8/9)	0% (0/9)	<0.001
+gonorrhea	44.4% (4/9)	0% (0/10)	.02
+chlamydia	11.1% (1/9)	10.0% (1/10)	.94
Urine protein (0-3+)	.7 (.7); 9	.3 (.7); 9	.33
Urine WBCs ≥10 cells/HPF (vs <10)	66.7% (6/9)	37.5% (3/8)	.23
Urine leukocyte esterase (0-3+)	1.4 (1.2); 8	.7 (1.2); 10	.24
Diagnosed with sepsis, % yes	11.1% (1/9)	0% (0/110	.26
Diagnosed with pelvic inflammatory disease, % yes	11.1% (1/9)	9.1% (1/11)	.88

THPO and gonorrhea, chlamydia, and/or trichomonas

There were 25.0% (12/48) women who received a positive test result for *N. gonorrhea*, *C. trachomatis*, and/or *T. vaginalis* (Table 6). Women with THPO and any STI, compared to those with THPO and no STI, were significantly younger and had higher urine leukocyte esterase (P<.05 for both).

**Table 6:** Women with THPO and Gonorrhea, Chlamydia, and/or Trichomonas Compared to Women with THPO and No Gonorrhea, Chlamydia, and Trichomonas Tubo-ovarian abscess, Hydrosalpinx, Pyosalpinx, and Oophoritis (THPO); Emergency Department (ED); Emergency Severity Index (ESI); White Blood Cells (WBC); High Power Field (HPF); Sexually Transmitted Infection (STI)

	+THPO and +STI (N=12)	+THPO and -STI (N=36)	P-value
Age (years)	26.0 (4.4); 12	34.3 (8.5); 36	<0.001
Black/African American race, %	91.7% (11/12)	86.1% (31/36)	.61
Arrived to ED by EMS/police, %	25.0% (3/12)	11.1% (4/36)	.24
ESI (0-5)	2.9 (.3); 12	3.0 (.2); 35	.54
Triage pain scale (0-10)	4.7 (3.6); 7	6.4 (2.8); 23	.28
Vaginal wet prep WBCs >10/HPF (vs ≤10)	58.3% (7/12)	32.3% (10/31)	.12
Vaginal wet prep clue cells, % present	50.0% (6/12)	33.3% (9/27)	.32
Vaginal wet prep yeast cells, % present	0% (0/11)	4.0% (1/25)	.50
Urine protein (0-3+)	.8 (.8); 12	.6 (.9); 33	.43
Urine WBCs/HPF	37.5% (9/24)	66.7% (8/12)	.10
Urine leukocyte esterase (0-3+)	1.4 (1.0); 11	.6 (1.0); 32	.046
Diagnosed with sepsis, % yes	0% (0/36)	8.3% (1/12)	.08
Diagnosed with pelvic inflammatory disease, % yes	8.3% (3/36)	16.7% (2/120	.41

## Discussion

Among women undergoing urinalysis and STI testing in the ED the diagnosis of THPO was made infrequently in only 0.3% of patient encounters. Patients diagnosed with THPO were majority Black/African-American and significantly more likely to be older than those without THPO. Interestingly, however, admitted THPO patients were statistically more likely to be younger than their discharged THPO counterparts. Patients with THPO were significantly more likely to be infected with gonorrhea but not chlamydia or trichomonas. Women in our dataset with THPO were also more likely to be diagnosed with sepsis and PID.

Previous reports suggest that TOA can be associated with PID in up to one-third to one-half of patients [5,13]. Based on these studies, we surmised that potentially high rates of STIs would be observed in our dataset among THPO patients. Contrary to this, however, among the 56 patients in our dataset, any STI (gonorrhea, chlamydia, and/or trichomonas) was present in only 12 cases (25%). In some instances, severe pelvic infections (including the entities of THPO) have been shown to result from bacteria typically found in the normal vaginal flora [14-15]. This might explain lower than presumed rates of STIs observed in our cohort of THPO patients.

TOA and THPO can be caused by pathogens other than the STIs examined in our study or even normal vaginal flora. For example, *Candida albicans* [7], *Streptococcus constellatus* [16], *Enterobius vermicularis* [17], *Salmonella* spp [18], *Brucella* spp [19], *Eikenella corrodens* [20], and *Actinomyces* spp [21] have all been implicated as causative microorganisms, but instances such as these are rarely reported. THPO infections, and in particular TOA, are often a combination of facultative, aerobic, and anaerobic bacteria [22]. TOA can also occur secondarily in the setting of surgical diseases such as inflammatory bowel disease, appendicitis, or diverticulitis [22]. The frequent polymicrobial nature of these severe pelvic infections may explain the findings in our dataset of relatively low STI rates in this patient population.

Consultation with specialists, need for advanced imaging or interventional procedures, and the inpatient process of obtaining a medical or surgical bed are all factors that may increase the duration of a patient’s ED clinical encounter with THPO [23]. We observed over a seven-fold increase in the duration of the encounter in THPO patients. Furthermore, of our 56 THPO patients, there was an even split between those requiring hospitalization versus those being deemed stable for discharge. Other than younger age, THPO patients who were admitted were not found to have statistically higher rates of sepsis, PID, or other laboratory derangements. These findings suggest that other serum markers or radiographic findings were likely the compelling factors driving admission in these individuals. Based on our dataset, however, we are unable to comment further on specific admission criteria.

Patients in our dataset with THPO and STIs tended toward co-infection with two or more STI organisms. This is consistent with prior findings in the literature suggestive that patients should be routinely screened for co-infection during STI testing [24-25]. Also, women in our cohort with THPO and any STI were significantly younger than THPO individuals without these infections. This is in line with the Centers for Disease Control and Prevention STI surveillance data, indicative of highest STI rates in young adults aged 20-24 years [26].

Limitations

Data are from a single health system in northeast Ohio. While a large number of women were tested for STIs, THPO was infrequently diagnosed in the dataset. The dataset did not include the results of imaging studies or markers of infection such as a complete blood count, serum lactate, C-reactive protein, or erythrocyte sedimentation rate. Data abstraction did not allow for delineation of final diagnosis, e.g. tubo-ovarian abscess versus pyosalpinx versus both. Furthermore, no pediatric patients (less than 18 years of age) were included in the dataset. Finally, not all women undergoing vaginal wet prep had a *T. vaginalis* NAAT performed, suggesting a degree of selection bias.

## Conclusions

THPO is an infrequently encountered entity in the ED, with 0.3% prevalence in our large dataset. Although an STI was identified in only 25% of THPO cases, patients with THPO were statistically more likely to be infected with gonorrhea than non-THPO counterparts. Furthermore, patients with THPO tended to be older and more frequently have a diagnosis of PID and sepsis compared with those individuals without this diagnosis. Although an uncommon diagnosis, ED providers must be attentive to patients presenting with pelvic symptoms that could be consistent with THPO to mitigate any complications that may arise and to direct the appropriate treatment.
